# Giant hemophilic pseudotumor eroding the iliac bone

**DOI:** 10.1093/omcr/omab005

**Published:** 2021-03-08

**Authors:** Bishesh S Poudyal, Gentle S Shrestha

**Affiliations:** 1 Clinical Haematology and Bone Marrow Transplant Unit, Government of Nepal, Civil Service Hospital, Minbhawan, New Baneshwor, Kathmandu, Nepal; 2 Department of Anaesthesiology, Tribhuvan University Teaching Hospital, Maharajgunj, Kathmandu, Nepal

A 22-year-old male, diagnosed with moderate hemophilia A (Factor VIII activity 2%) at the age of 2 years, presented with painful spontaneous swelling of right groin. In the previous 6 months, he was admitted twice with right psoas muscle bleed. Computed tomography (CT) scan revealed 5 cm × 7 cm heterogeneous collection in right iliopsoas muscle. Patient was treated with fresh frozen plasma, tranexamic acid and recombinant factor VIII. Because of repeated episodes of bleeding, he was advised for Factor VIII prophylaxis. He could not comply because of cost constraints.

He presented 2 years later with large swelling (6 cm × 5 cm) over the right groin. CT scan of the abdomen showed 27.0 cm × 12.6 cm × 9.8 cm mixed density mass eroding the right iliac blade (Fig. 1a) with subcutaneous collection in right gluteal region (Fig. 1b and Figure supplement). The fine needle aspiration revealed hematoma, which was suggestive of hemophilic pseudotumor. It is a rare complication of hemophilia presenting with progressive cystic swelling of muscle and/or bone due to repeated bleeding, occurring in less than 2% of hemophiliacs and up to 10% in those with inhibitors [1]. Radiographic findings vary greatly with the extent, location, and stages of hemorrhages and reflect the presence of medullary bone destruction, cortical changes, internal opacities, various types of periosteal reaction and surrounding soft-tissue abnormalities [2]. Management of hemophilic pseudotumor includes conservative methods, radical extirpation and irradiation [3]. Surgical excision is recommended for large pelvic and abdominal pseudotumors [4]. The inhibitor assay of the patient was six Bethesda units. Nearly 30% of hemophilia A patients develop inhibitor against factor VIII [5]. Inhibitor usually develops within 20 exposure days [6]. Our patient had received factor VIII 12 times.

**Figure 1:**
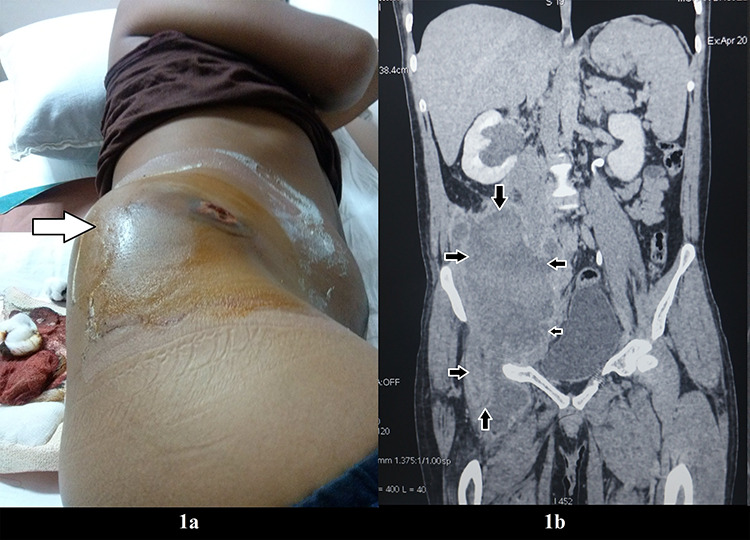
(**a**) Firm mass in the right groin (indicated by arrow); (**b**) CT scan of the abdomen showing a large mixed density pelvic mass eroding the right iliac blade with extension to the outer surface of the iliac blade (delineated by small arrows).

Surgery was performed using Factor VII concentrate at standard dose (90 mcg/kg 2 hourly for 48 hours, then 90 mcg/kg 4 hourly for next 48 hours and then 6 hourly for next 72 hours). He was then administered activated prothrombin complex (FEIBA) 50 IU/kg twice a day for 7 days. He was discharged on day 14 and was kept on factor eight inhibitor bypass activity prophylaxis at 50 IU/kg every alternative days for 1 week and then twice a week. Three months after surgery, patient was asymptomatic and was back to usual activities.

## Supplementary Material

Figure_supplement_omab005
