# Supplementary figures and images for: Giant hemophilic pseudotumor eroding the iliac bone
Source: Oxf Med Case Reports. 2021 Mar 8;2021(3):omab005. doi: 10.1093/omcr/omab005 (PMC7947266; doi:10.1093/omcr/omab005)

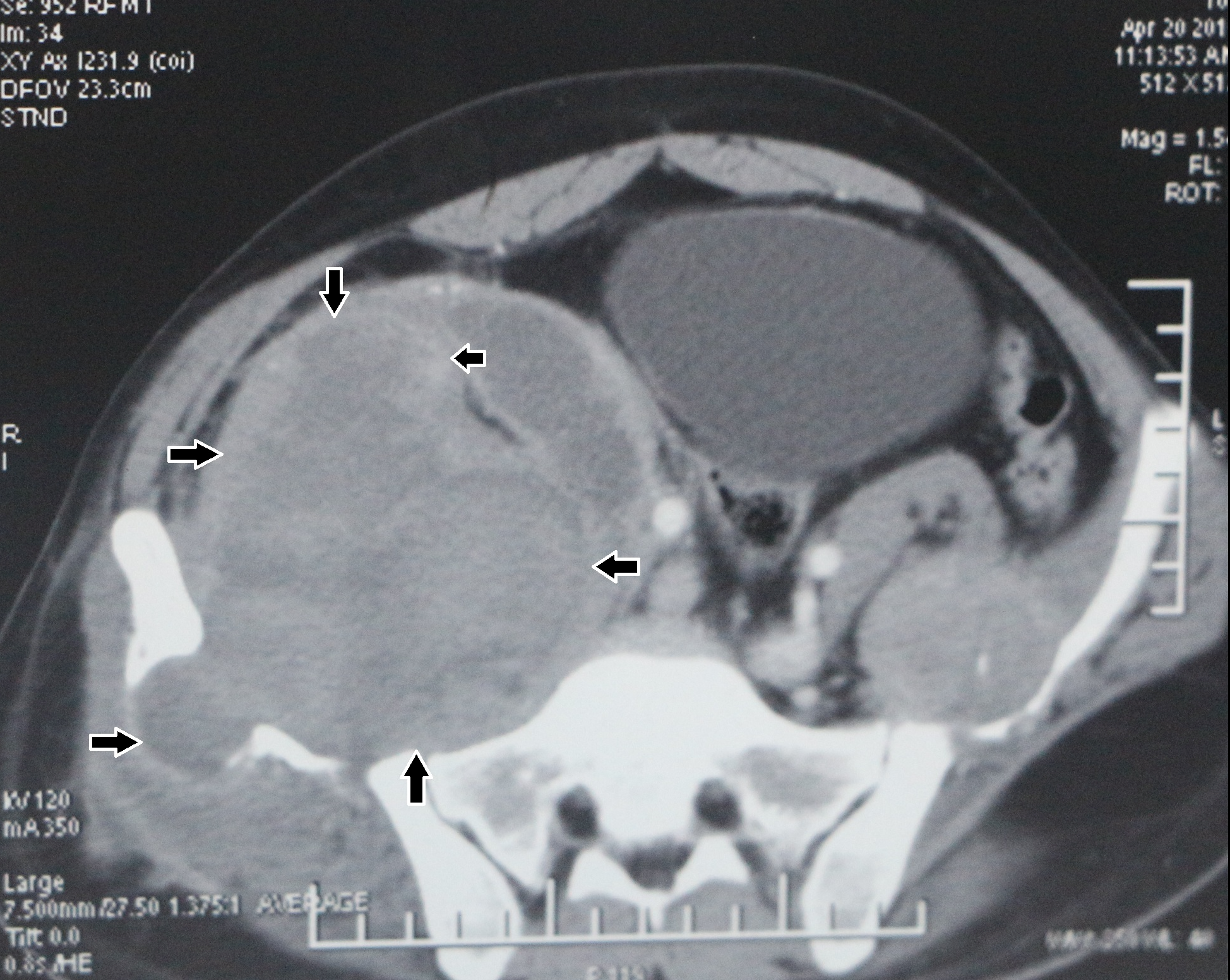

Supplement: Figure_supplement_omab005 [file figure_supplement_omab005.png]
